# Serial Changes of Long COVID Symptoms and Clinical Utility of Serum Antibody Titers for Evaluation of Long COVID

**DOI:** 10.3390/jcm11051309

**Published:** 2022-02-27

**Authors:** Yasue Sakurada, Naruhiko Sunada, Hiroyuki Honda, Kazuki Tokumasu, Yuki Otsuka, Yasuhiro Nakano, Yoshihisa Hanayama, Masanori Furukawa, Hideharu Hagiya, Fumio Otsuka

**Affiliations:** 1 Department of General Medicine, Okayama University Graduate School of Medicine, Dentistry and Pharmaceutical Sciences, Okayama 700-8558, Japan; sakurada202@gmail.com (Y.S.); naru.kun.red.1117@gmail.com (N.S.); ppgf1hrd@okayama-u.ac.jp (H.H.); tokumasu@okayama-u.ac.jp (K.T.); otsuka@s.okayama-u.ac.jp (Y.O.); y-nakano@okayama-u.ac.jp (Y.N.); hanayama@md.okayama-u.ac.jp (Y.H.); hagiya@okayama-u.ac.jp (H.H.); 2 Department of Laboratory Medicine, Okayama University Hospital, Okayama 700-8558, Japan; furuka-m@okayama-u.ac.jp

**Keywords:** Anti-SARS-CoV2 antibody, dysgeusia, dysosmia, general fatigue, long COVID

## Abstract

Background: Various symptoms persist even after the acute symptoms in about one third of patients with COVID-19. In February 2021, we established an outpatient clinic in a university hospital for patients with long COVID and started medical treatment for sequelae that persisted one month or more after infection. Methods: To determine the key factors that affect the onset and clinical course of sequelae, a retrospective analysis was performed at Okayama University Hospital (Japan) between February and July 2021. We focused on changes in the numbers of symptoms and the background of the patients during a three-month period from the first outpatient visit. We also examined the relationship with SARS-CoV-2 antibody titers. Results: Information was obtained from medical records for 65 patients. The symptoms of sequelae were diverse, with more than 20 types. The most frequent symptoms were general malaise, dysosmia, dysgeusia, sleeplessness, and headache. These symptoms improved in about 60% of the patients after 3 months. Patients who required hospitalization and had a poor condition in the acute phase and patients who received oxygen/dexamethasone therapy had higher antibody titers at the time of consultation. Patients with antibody titers ≥200 U/mL showed significantly fewer improvements in long COVID symptoms in 1 month, but they showed improvements at 3 months after the first visit. Conclusion: Long COVID symptoms were improved at 3 months after the initial visit in more than half of the patients. Serum antibody titers were higher in patients who experienced a severe acute phase, but the serum antibody titers did not seem to be directly related to the long-term persistence of long COVID symptoms.

## 1. Introduction

The novel coronavirus disease 2019 (COVID-19) pandemic is ongoing almost 2 years after the declaration of a pandemic by the World Health Organization (WHO) [1]. COVID-19 causes various symptoms after an incubation period of several days and can result in death in patients with risk factors such as advanced age, obesity, diabetes mellitus, hypertension, and allergic diseases [2,3,4,5,6,7,8,9].

In addition to acute-phase symptoms, COVID-19 can cause prolonged sequelae, which have recently been termed as long COVID or post-acute sequelae of SARS-CoV-2 infection (PASC) and have recently been defined as post-COVID-19 condition by WHO [10,11,12]. A wide variety of persistent conditions including general malaise, dysgeusia, dysosmia, low-grade fever, headache, and alopecia have been reported [11,13,14,15,16]. According to previous reports, at least one-third of COVID-19 patients have long COVID [17,18], and an effective treatment strategy for long COVID has not yet been established. Moreover, the long-term outcomes of long COVID as well as factors associated with its prognosis are still unclear. Since the pathophysiology of long COVID is not known and a therapeutic strategy for long COVID has not been established, general physicians may avoid seeing patients with long COVID because of the lack of sufficient guidelines for its management [19,20].

On 15 February in 2021, we established a COVID-19 aftercare clinic (CAC) in Okayama University Hospital (Japan). The costs of treatment for patients visiting the clinic are covered by National Health Insurance. The reasons for establishing the clinic were to provide treatment for patients suffering from long COVID and to analyze the clinical characteristics of patients with long COVID in order to establish treatment strategies. Our previous study revealed that (i) most patients with long COVID had not required hospitalization in the acute phase and (ii) general fatigue was a major complaint in more than half of the patients [21].

In the present study, we analyzed follow-up data over a 3-month period for patients with long COVID who visited our clinic, in order to determine how each of the sequelae deteriorated or improved. We also examined serum antibody titers in all of the patients to determine whether antibody titers can be a clue for estimating the prognosis of sequelae.

## 2. Patients and Methods

### 2.1. Patients’ Characteristics and Changes in Long COVID Symptoms

This study was a retrospective, observational study. Medical records for 65 patients who visited our CAC due to long COVID during the period from February to July in 2021 were carefully reviewed. Long COVID was defined as symptoms that persist for more than one month after the onset of COVID-19 [10,11,12]. Information was obtained from medical records for age, gender, body mass index (BMI), underlying conditions (current smoking and alcohol drinking habits), hospitalization due to COVID-19, therapeutic events of either oxygen or corticosteroid use during the acute phase, date of visiting the CAC after the onset of COVID-19, severity of COVID-19, history of COVID-19 vaccination, and clinical symptoms of long COVID. The deemed level of severity of COVID-19 complied with the Japanese classification defined by the Ministry of Health, Labour and Welfare in Japan, i.e., mild: without shortness of breath or pneumonia (percutaneous oxygen saturation: SpO_2_ ≥ 96%); moderate-I: with pneumonia but not respiratory failure (93% < SpO_2_ < 96%); moderate-II: with pneumonia accompanying respiratory failure and oxygen administration required; severe (SpO_2_ ≤ 93%): admission to an intensive care unit or artificial ventilation required [22].

The clinical data used in this study were derived from actual medical data from an outpatient clinic, and this is a major difference from other internet or questionnaire-based surveys. Patients were free to describe any of the symptoms they were having trouble with. The complete disappearance of symptoms was regarded as “no symptoms”, while the presence of any remaining symptoms was regarded as “symptoms”. Therefore, the severity of long COVID symptoms was not included in the evaluation, whereas the number of long COVID symptoms was carefully incorporated in the study. The presence of clinical symptoms was first screened at the initial visit and then followed up every month until the third month in each medical interview.

### 2.2. Analysis of Antibody Titers

Examinations of serum antibodies were used by each physician to evaluate the past infection condition and/or the possibility of vaccination. Serum samples obtained at initial visits were used for measurement of anti-SARS-CoV-2 antibody using the Elecsys Anti-SARS-CoV-2 S (S300) electrochemiluminescence (ECLIA) kit (Roche Diagnostics, Rotkreuz, Switzerland) and cobas 8000 modular analyzer system at the Central Laboratory of Okayama University Hospital. The measurement range is from 0.40 U/mL to 25,000 U/mL (with 1:100 dilution) with a concentration of <0.80 U/mL considered negative. Regarding the unit exchanges of the antibody titer, since the unit value derived from this ECLIA kit has the highest linearity range to the binding antibody unit (BAU) based on the WHO International Standard [23] for COVID-19 serological tests (Elecsys IU = 0.972 × BAU: *R* = 0.9996), the present unit “U/mL” substantially represents “BAU/mL”. The thresholds of the antibody titers were set to 50, 100, 200, and 500 U/mL to stratify the patients based on a past report [24].

### 2.3. Statistical Analysis

EZR, version 1.40 (Saitama Medical Center, Jichi Medical University, Saitama, Japan), which is a graphical user interface for R (The R Foundation for Statistical Computing, Vienna, Austria), was used in all statistical analyses [25]. It is modified from R commander, which is designed to add frequently used functions in biostatistics. Fisher’s exact test and the Mann-Whitney U test were used to compare two categorical variables, and Spearman’s rank correlation coefficient was used to statistically analyze continuous measurements. All tests were performed as two-sided, and * *p* < 0.05 and ** *p* < 0.01 were regarded as statistically significant.

## 3. Results

During the study period, 65 patients visited our CAC. Of these, 9 patients, 12 patients, and 15 patients did not visit the clinic in the first month, second month, and third month, respectively, after the initial visit. The clinical backgrounds of the patients with long COVID are shown in Table 1. The 65 patients included 29 males (44.6%) and 36 females (55.4%). The median age of all the patients was 39 years (interquartile range [IQR]: 25–54 years). The median age of the male patients was 39 years (IQR: 23–50 years) and that of the female patients was also 39 years (IQR: 28–56 years). The median BMI of all patients was 22.0 (IQR: 20.2–26.2) and the median BMIs of the male and female patients were 24.7 (IQR: 21.8–27.0) and 21.1 (IQR: 19.6–24.6), respectively (male vs. female: *p* = 0.0163). There were 28 patients (43.1%) with a smoking habit, 28 patients (43.1%) with a drinking habit, 22 patients (33.8%) with admission due to COVID-19, and 11 patients (16.9%) who had received oxygen/steroid therapy. The median duration from onset of COVID-19 to visiting the clinic was 73 days (IQR: 54–114 days). The duration was less than 60 days for 23 patients (35.4%) and the duration was more than 60 days for 42 patients (64.6%). As for the severity of COVID-19 defined by the Ministry of Health, Labour and Welfare in Japan [22], the numbers (proportions) of patients with mild, moderate-I, moderate-II, and severe states were 50 (76.9%), 4 (6.2%), 8 (12.3%), and 3 (4.6%), respectively.

**Figure 1:**
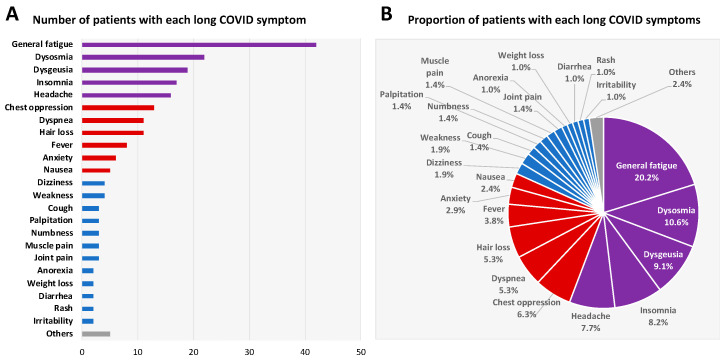
Number of patients and proportion of patients with each long COVID symptom at the initial visit. Number of the patients (**A**) suffering from each symptom in the 65 patients and the proportion of patients (**B**) with each symptom of long COVID are shown. The 5 most frequent symptoms were general fatigue, dysosmia, dysgeusia, insomnia, and headache (purple) and the next 5 most frequent symptoms were chest compression, dyspnea, hair loss, low-grade fever, anxiety, and nausea (red).

**Figure 2:**
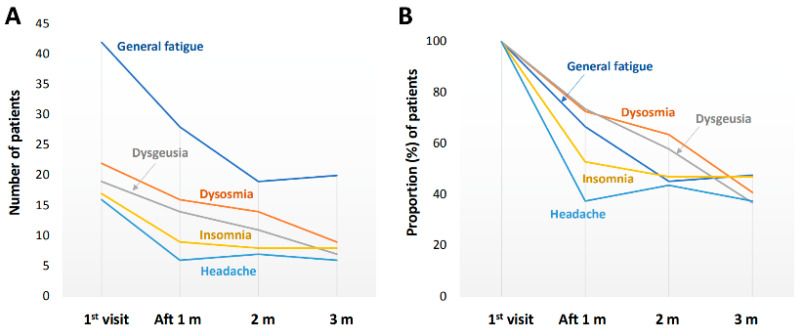
Improvements in the five most common symptoms of long COVID in the three-month follow-up period. Changes in (**A**) the number of patients and (**B**) the proportion (%) of patients who suffered from each symptom.

**Figure 3:**
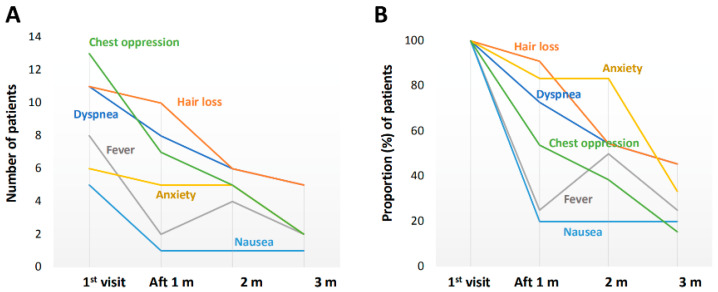
Improvements in other symptoms of long COVID during the three-month follow-up period. Changes in (**A**) the number of patients and (**B**) the proportion (%) of patients with each symptom.

The number of patients and the proportion of patients with each long COVID symptom at the initial visit are shown in Figure 1. There were more than 20 symptoms of long COVID. The five most frequent symptoms were general fatigue (42 patients, 20.2%), dysosmia (22 patients, 10.6%), dysgeusia (19 patients, 9.1%), insomnia (17 patients, 8.2%), and headache (16 patients, 7.7%). Improvements in the five most common symptoms of long COVID over time are shown in Figure 2. At 3 months after the initial visit, the numbers of patients with any remaining symptoms had decreased to about half of the numbers at the initial visit. The proportions of patients with general fatigue, dysosmia, dysgeusia, insomnia, and headache at 3 months were 47.6%, 40.9%, 36.8%, 47.1%, and 37.5%, respectively (Figure 2). The proportions of patients with symptoms of chest opression, dyspnea, hair loss, low-grade fever, anxiety, and nausea were 15.4%, 45.5%, 45.5%, 25.0%, 33.3%, and 20.0%, respectively (Figure 3).

**Figure 4:**
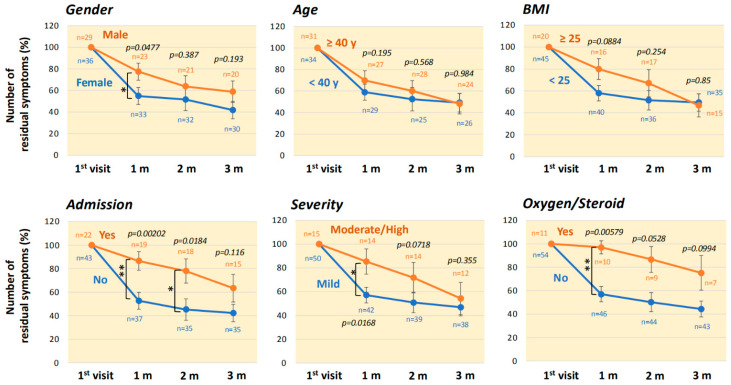
Serial changes in the numbers of residual symptoms in the three-month period according to clinical backgrounds. Changes in the numbers of residual symptoms of long COVID during the three-month period according to patients’ backgrounds and clinical factors related to COVID-19. The data are shown as means ± SEM and were analyzed by the Mann-Whitney U test: ** *p* < 0.01 and * *p* < 0.05, statistically significant between the indicated factors (“*n*” indicates the number of patients).

Changes in the numbers of any remaining symptoms during the 3-month period according to clinical backgrounds are shown in Figure 4. The number of symptoms remaining one month after the initial visit in male patients was significantly larger than that in female patients (*p* = 0.0477), but there was no significant difference between male and female patients at three months. When compared by age categories based on the distribution and median of ages (Table 1), the number of residual symptoms for three months after the initial visit was not significantly different between patients aged <40 years and patients aged ≥40 years. The number of residual symptoms at one month after the initial visit tended to be larger for obese patients with BMI of ≥25 *(p* = 0.0884), but there was no difference between the BMI categories at three months. The number of residual symptoms was significantly larger for patients who required hospitalization in the acute phase both at one month (*p* = 0.00202) and two months (*p* = 0.0184). The number of residual symptoms at one month was significantly larger for moderate and severe cases than for mild cases (*p* = 0.0168). The number of residual symptoms was significantly larger for patients who received oxygen/corticosteroid therapies in the acute phase, especially at the first month after the initial visit (*p* = 0.00579).

**Figure 5:**
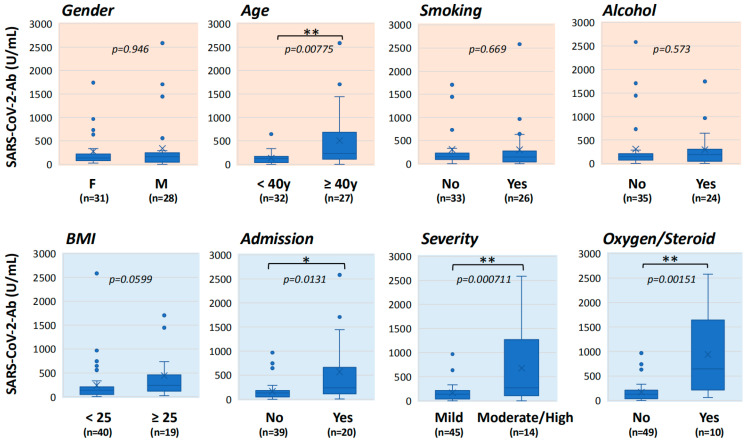
Differences of serum antibody titers based on the clinical background. Serum anti-SARS-CoV-2 antibody levels of the long COVID patients at the initial visit were compared between two groups based on the patients’ backgrounds and clinical factors related to COVID-19. In each panel, the upper horizontal line of the box is the 75th percentile, the lower horizontal line of the box is the 25th percentile, the horizontal bar within the box is the median, the upper horizontal bar outside the box is the maximum value within 1.5 times of the interquartile range, and the lower horizontal bar outside the box is the minimum value within 1.5 times of the interquartile range. The data were analyzed by the Mann-Whitney U test: ** *p* < 0.01 and * *p* < 0.05, statistically significant between the indicated factors (“*n*” indicates the number of patients).

Serum anti-SARS-CoV-2 antibody levels in the long COVID patients at the initial visit were compared in each category: gender (females and males), age (<40 years and ≥40 years), history of smoking and alcohol, BMI (<25 and ≥25), hospitalization, severity in the acute phase, and use of oxygen or corticosteroid therapy (Figure 5). Five patients with a history of COVID-19 vaccination and one patient who refused to undergo a blood examination were excluded. There were no significant differences in antibody titers in the categories of gender, BMI, smoking, and alcohol consumption. The antibody titer was significantly higher in patients aged ≥40 years (228 U/mL [IQR: 108–681 U/mL]) than in patients aged <40 years (120 U/mL [IQR: 38–166 U/mL]) (*p* = 0.00775), and the antibody titer was significantly higher in patients who were hospitalized in the acute phase (236 U/mL [IQR: 113–657 U/mL]) than in patients who were not hospitalized in the acute phase (132 U/mL [IQR: 46–178 U/mL]) (*p* = 0.0131). The antibody titer was also significantly higher in patients with severe or moderate acute symptoms (424 U/mL [IQR: 168–1269 U/mL]) than in patients with mild symptoms (132 U/mL [IQR: 33.5–204 U/mL]) (*p* = 0.000711), and it was also significantly higher in patients who received oxygen and/or corticosteroid therapies (644 U/mL [IQR: 217–1642 U/mL]) than in patients who did not receive such therapies (134 U/mL [IQR: 40–209 U/mL]) (*p* = 0.00151).

**Figure 6:**
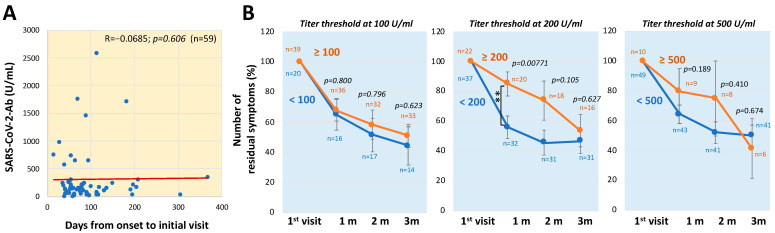
Serial changes in the number of residual symptoms during the three-month period after the initial visit according to antibody titers. (**A**) Interrelationship between serum antibody levels and duration from disease onset to initial visit. Spearman’s rank correlation coefficient was used for statistical analysis. (**B**) Changes in the numbers of residual symptoms of long COVID during the three-month period were compared between two groups based on the titers of anti-SARS-CoV-2 antibody in the patients’ sera. The data are shown as means ± SEM and were analyzed by the Mann-Whitney U test: ** *p* < 0.01, statistically significant between the indicated factors (“*n*” indicates the number of patients).

In addition, to determine the influence of timing from the onset of COVID-19 until the hospital visit on the serum antibody level, the interrelationship between the number of days from the onset to the initial visit and serum anti-SARS-CoV-2 antibody titers at the initial visits were analyzed. As shown in Figure 6A, some patients had a relatively high serum antibody titer; however, there was no significant interrelationship between the length of time before the visit and the serum antibody level (*R* = −0.0685; *p* = 0.606; *n* = 59). Furthermore, changes in the proportions of residual symptoms for three months after the initial visit were compared at the cut-off levels of 100, 200 and 500 U/mL (Figure 6B). In the titer-dependent groups, the number of residual symptoms was significantly larger for patients with serum titers of ≥200 U/mL at one month (*p* = 0.00771), but there was no difference between the numbers of residual symptoms in patients with serum titers of ≥200 U/mL and patients with serum titers of <200 U/mL at two or three months.

## 4. Discussion

The patients with long COVID suffered from a large variety of symptoms (more than 20 symptoms). The number of patients with residual long COVID symptoms at the first month after the initial visit was significantly larger for patients who had been hospitalized, especially moderately or severely ill patients, and patients who required oxygen/corticosteroid therapy. However, the five most common sequelae had subsided at 3 months after the initial visit in more than half of the patients. The serum antibody titer was significantly higher in patients who experienced a severe acute phase, but it did not appear to be associated with a long-term prognosis of residual symptoms.

In this study, we obtained information for patients with long COVID directly through medical interviews and by physical examinations at our CAC. Studies on long COVID in Europe, the United States, China and Japan, [21,26] as well as a meta-analysis have gradually revealed the clinical characteristics and prognosis of long COVID [13,27]. However, most of the information for those studies was obtained from online or telephone interviews and there was no follow-up of the patients in those studies. The information obtained for this study shows the symptoms of patients in detail and would thus be of great value for physicians who see long COVID patients at real outpatient settings.

Our patient database included almost the same numbers of men and women, and their backgrounds did not differ significantly [28]. There does not seem to be a fundamental difference in the major symptoms of long COVID between male and female patients (Supplementary Figure S1). However, general fatigue was a more frequent symptom in male patients than in female patients and there was a tendency for general fatigue to persist for three months unlike other residual symptoms such as dysosmia, dysgeusia, sleeplessness and headache in male patients (Supplementary Figure S2). On the other hand, complaints of hair loss and dyspnea were more frequent in female patients than in male patients (Supplementary Figure S1).

According to previous reports [29,30,31], risk factors for prolonged sequelae include age, gender, BMI, disease severity, and number of symptoms. Our data also suggested that history of hospitalization, severity of the acute phase and oxygen/corticosteroid therapy are associated with persistence of long COVID symptoms. However, significant differences were observed only at the first month after the initial visit, and the differences gradually disappeared over the three-month period.

A notable point of our study is that we investigated the associations between serum antibody titers and long COVID symptoms. As expected, serum antibody titers were significantly higher in patients with severe acute conditions. The number of persistent symptoms was significantly larger for patients with higher antibody titers (≥200 U/mL) at the initial visit; however, the difference in residual symptoms disappeared by the third month. Regarding the cut-off value of the antibody level, when groups with anti-SARS-CoV-2 antibody titers below and above the cut-off levels 50, 100 and 500 U/mL at the first visit were compared, there was no significant difference in the rates of residual symptoms. This result suggests that measurement of serum antibody titers in patients suffering from long COVID may not provide simple guidance as to the persistence of prolonged symptoms.

In this regard, Garcia-Abellan and colleagues revealed the critical factors for prediction of long COVID after hospitalization for COVID-19 in a prospective study on 146 patients [32]. In their study, the predictors for prolonged symptoms persisting for from 2 to 6 months were lower peak anti-SARS-CoV-2-antibody, higher WHO severity score, and female sex [32]. In contrast, in our study, the rate of reduction in the number of long COVID symptoms was transiently delayed in patients with severe COVID-19 in the acute phase, which was defined by histories of hospitalization, oxygen/steroid therapy, and serum high titers of anti-SARS-CoV-2-antibody. Thus, a low SARS-CoV-2 antibody titer in the acute phase is likely to be a risk factor for prolonged symptoms of long COVID [14,32], whereas a high titer of the antibody may also cause a delay in the reduction of various long COVID symptoms. Further studies are needed to determine the usefulness of measurement of antibody titers for prognosis of long COVID symptoms.

We should also note limitations of this study. First, we did not have detailed information on the acute-phase treatments for patients. Treatment during the acute phase of COVID-19 may influence the degrees of clinical manifestations of long COVID. Second, we followed up the patients for only 3 months and further long-term observation is needed to clarify how long the symptoms persist. Third, because of the limited number of patients, all possible symptoms of long COVID might not have been included. Also, we could not sufficiently stratify or adjust severity and patients’ backgrounds for estimating the improvement of each manifestation. Fourth, in the present study, the duration between the onset of COVID-19 and the first visit was not apparently involved in the serum antibody titer at the first visit. However, the various durations from disease onset to the initial visit could also have influenced the trends in changes of the symptoms. Fifth, we set the cut-off level of the serum antibody titers to from 50 to 500 U/mL according to a previous report without any validation study [24] in the current situation in which no clear antibody titer level has been established for evaluating long COVID manifestations. Further investigation on the relationships between clinical manifestations and antibody titers in patients with long COVID should be performed. Despite these limitations, our clinical data provide indicators for future improvements in long COVID management.

In summary, we found that common long COVID symptoms improved within 3 months after the initial visit to our clinic in more than half of the patients, though the sequelae of COVID-19 are very diverse. One of the merits of our study is the detailed collection of information on patients’ symptoms based on face-to-face medical interviews at each visit, and this information is more reliable than information obtained from simplified questionnaires. Serum antibody titers were higher in patients who experienced a severe acute phase, but they did not seem to be directly related to reduction of the number of long COVID symptoms. A relatively large percentage of patients infected with SARS-CoV-2 suffer from long COVID, which has long-term adverse effects on their social life. Multifaceted approaches for establishing methods for evaluation and prediction of prognosis and for establishing treatment strategies are needed for the management of long COVID.

## Figures and Tables

**Table 1:** Backgrounds of patients visiting the COVID-19 aftercare outpatient clinic (*n* = 65). IQR, interquartile range; the data were analyzed by (a) by Mann-Whitney U or (b) Fisher’s exact tests; and * *p* < 0.05, statistically significant.

	Total	Male	Female	*p* Value
No. of patients, by gender	65	29 (44.6%)	36 (55.4%)	
Age, years, median (IQR)	39 (25, 54)	39 (23, 50)	39 (28, 56)	0.265 (a)
BMI (IQR)	22.0 (20.2, 26.2)	24.7 (21.8, 27.0)	21.1 (19.6, 24.6)	* 0.0163 (a)
Clinical underlying conditions, n (%)				
Smoking habit	28 (43.1%)	16 (55.2%)	12 (33.3%)	0.0861 (b)
Alcohol drinking habit	28 (43.1%)	15 (51.7%)	13 (36.1%)	0.221 (b)
Admission due to COVID-19	22 (33.8%)	12 (41.4%)	10 (27.8%)	0.298 (b)
Oxygen/Steroid therapy	11 (16.9%)	6 (20.7%)	5 (13.9%)	0.52 (b)
Duration after onset to visit, median (IQR)	73 days (54, 114): <60 days, 23 cases (35.4%); ≥60 days, 42 cases (64.6%)			
Severity of COVID-19	Mild, 50 (76.9%); Moderate-I, 4 (6.2%); Moderate-II, 8 (12.3%); Severe, 3 (4.6%)			

## Data Availability

Information regarding the present study was provided on our hospital wall and on the website of our hospital, and patients who wished to opt out were offered that opportunity.

## Supplementary Materials

The following supporting information can be downloaded at: https://www.mdpi.com/article/10.3390/jcm11051309/s1. Figure S1: Number of female patients and number of male patients with each long COVID symptom at the initial visit; Figure S2: Improvements in the 5 most common symptoms of long COVID during the 3-month follow-up period in female and male patients.
