# Supplementary material for: Serial Changes of Long COVID Symptoms and Clinical Utility of Serum Antibody Titers for Evaluation of Long COVID
Source: J Clin Med. 2022 Feb 27;11(5):1309. doi: 10.3390/jcm11051309 (PMC8911256; doi:10.3390/jcm11051309)
Supplement: Supplementary file 1 [file jcm-11-01309-s001.zip › jcm-1589624-supplementary.pdf]

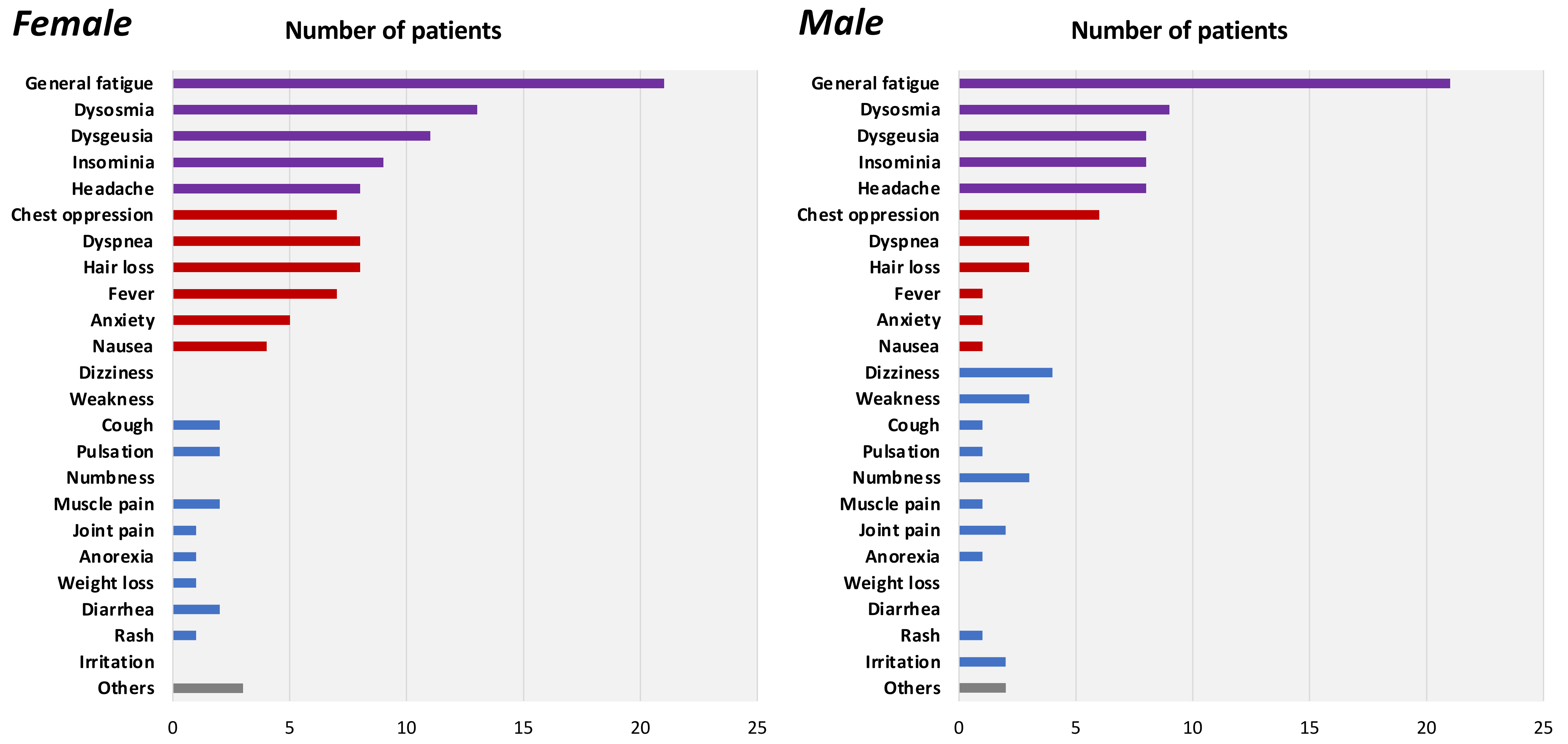

Figure S1. Number of female patients and number of male patients with each long COVID symptom at the initial visit.

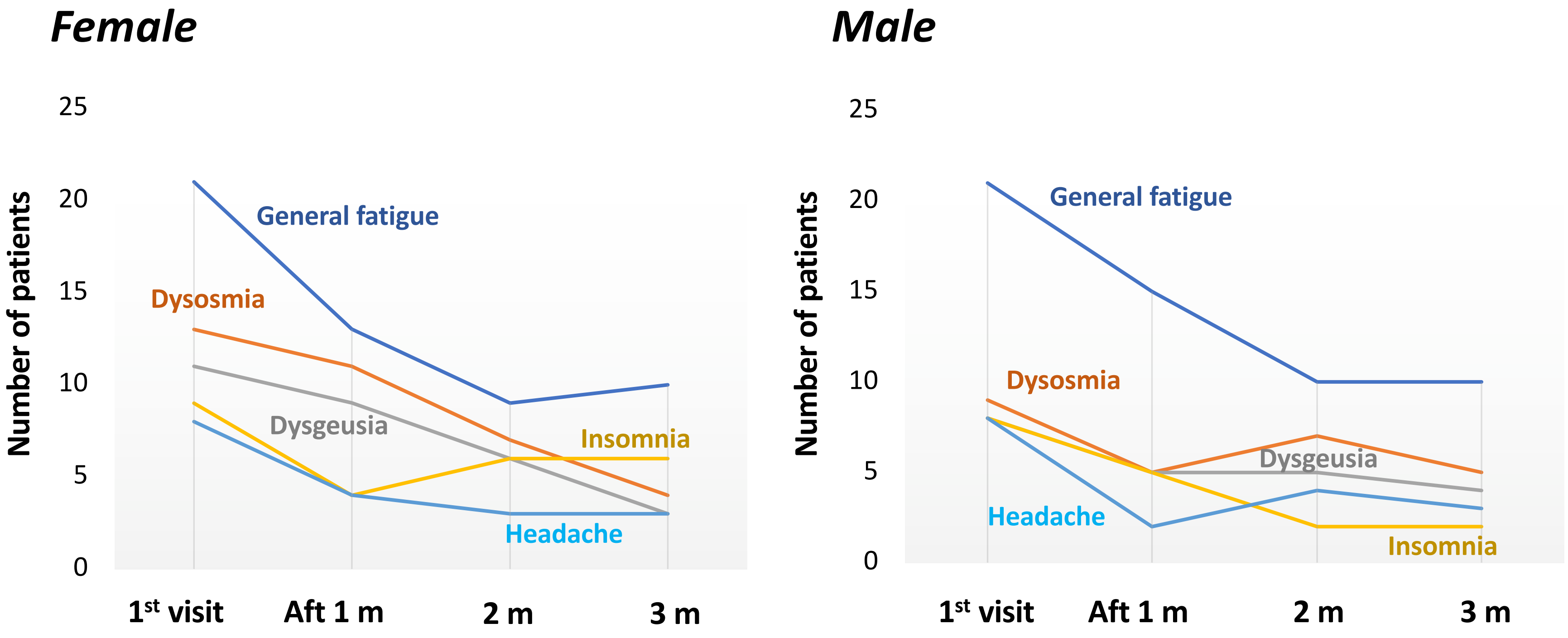

Figure S2. Improvements in the 5 most common symptoms of long COVID during the 3-month follow-up period in female and male patients.
